# Usage of an Exercise App in the Care for People With Osteoarthritis: User-Driven Exploratory Study

**DOI:** 10.2196/mhealth.7734

**Published:** 2018-01-11

**Authors:** Dorthe Boe Danbjørg, Allan Villadsen, Ester Gill, Mette Juel Rothmann, Jane Clemensen

**Affiliations:** ^1^ Centre for Innovative Medical Technology Department of Clinical Research University of Southern Denmark, Odense University Hospital Odense Denmark; ^2^ Quality of Life Research Center Department of Haematology Odense University Hospital Odense Denmark; ^3^ Orthopaedic Research Unit Department of Orthopaedic Surgery and Traumatology University of Southern Denmark, Odense University Hospital Odense Denmark; ^4^ Department of Clinical Research University of Southern Denmark Odense Denmark; ^5^ Research Unit of Medical Endocrinology Department of Endocrinology University of Southern Denmark, Odense University Hospital Odense Denmark; ^6^ Research Unit of Rheumatology Department of Rheumatology University of Southern Denmark, Odense University Hospital Odense Denmark; ^7^ Research Unit of Paediatrics Hans Christian Andersen Children's Hospital University of Southern Denmark, Odense University Hospital Odense Denmark

**Keywords:** arthritis, rehabilitation, telemedicine

## Abstract

**Background:**

Exercise has proven to reduce pain and increase quality of life among people living with osteoarthritis (OA). However, one major challenge is adherence to exercise once supervision ends.

**Objective:**

This study aimed to identify mental and physical barriers and motivational and social aspects of training at home, and to test or further develop an exercise app.

**Methods:**

The study was inspired from participatory design, engaging users in the research process. Data were collected through focus groups and workshops, and analyzed by systematic text condensation.

**Results:**

Three main themes were found: competition as motivation, training together, and barriers. The results revealed that the participants wanted to do their training and had knowledge on exercise and pain but found it hard to motivate themselves. They missed the observation, comments, and encouragement by the supervising physiotherapist as well as their peers. Ways to optimize the training app were identified during the workshops as participants shared their experience.

**Conclusions:**

This study concludes that the long-term continuation of exercising for patients with OA could be improved with the use of a technology tailored to users’ needs, including motivational and other behavioral factors.

## Introduction

### Background

Osteoarthritis (OA) is a degenerative joint disease that causes pain and decreases physical function and quality of life. It is the most common musculoskeletal disorder [1,2], and globally, it is a heavy economic burden [3] with annual costs of US $89.1 billion in the US alone [4].

Exercise reduces pain at the same level as simple analgesics and nonsteroidal anti-inflammatory drugs for people with OA of the hip and knee [5]. Furthermore, it increases physical function and quality of life [6]. Hence, exercise is considered one of the cornerstones in the treatment of hip and knee OA [6-9]. General recommendations are to offer OA patients information or education of the different aspects of the disease in conjunction with supervised exercise for 6 to 12 weeks [10,11]. Exercise is encouraged to be continued lifelong. In Scandinavia, this approach is generally accepted by both patients and health professionals but tends to be an underutilized treatment option among medical practitioners [12-14].

A dose-response relationship has been demonstrated between adherence to exercise and effect on people with knee OA [15]. Thus, the effectiveness of exercise on pain relief and disability only lasts as long as the patient participates [16,17]. However, when prescribed or allocated to exercise therapy, one major challenge seems to be adherence to exercise once supervision ends [18]. There may be many individual barriers to exercise—for example, busy daily schedules and lack of motivation. Accessible technologies may, to some extent, address these barriers. The percentage of the population who owns a smartphone or a desktop computer and has access to the Internet is rising and includes the elderly age group. Hence, an information technology solution may be able to reach a wide population. Furthermore, it may have economic benefits because treating more patients will not necessarily require more hours from physicians or other health care professionals [19].

### Internet-Based Training

Internet-based training concepts aiming to improve the exercise level among chronically ill patients, such as patients with hip and knee OA, have already been developed and tested, but it is still essential to identify features in these exercise apps that will lead to sustained long-term usage [20,21]. An individualized approach to exercise is deemed essential for an optimal effect in OA treatment [18]. With the use of on a Web-based training app, it would be possible to personalize the exercise program and individualize motivational factors, which may optimize an individual’s outcomes. Some studies show that class-based exercise is more effective in regard to adherence. Group interaction could potentially be added to the exercise app’s interface, which generally is customized, to fit the personal needs of the individual user [5,21].

In 2014, an exercise app (Therapeutical exercise [Ther-ex]) (Ther-Ex APS, Denmark) targeting people with OA was brought to market in both iOS and Android versions. The developers were an orthopedic surgeon and a physiotherapist. Their concept for the app was to compile general OA recommendations of exercise and its monitoring into a solution, which was readily available for people with OA. The app contains approximately 100 individual videos of land-based functional exercises, which can be combined into individualized exercise programs (Textbox 1). Furthermore, the app contains an exercise and pain rating log, and these data can be displayed in various ways.

### Aim

Limited user feedback on the app has generally been positive. However, troublesome functions have been identified when the app was tested in 2014. The current content was found insufficient to support adherence to exercise. To solve these problems and improve the app and the resultant self-care for people with OA, a systematic approach based on user participation was chosen.

The aim of this study was threefold:

1. To identify the mental and physical barriers and motivational and social aspects of training at home for people with the hip or knee arthritis
2. To test an exercise app for use at home by patients with hip or knee OA
3. To enhance the app on the basis of users’ experiences with physical barriers, their motivational and social aspects of training, and their experiences with using the app

**Exercise app—therapeutical exercise.**

Idea: to compile general osteoarthritis (OA) recommendations of exercise and its monitoring into an easy accessible tool

Contains: approximately 100 videos of land-based functional exercises and combines these into the following:

- Exercise programs
- Visual pain rating scale
- Exercise and weight diary
- Module for visualization of the above

Developed by a physiotherapist and an orthopedic surgeon with both clinical and research knowledge of OA

## Methods

### Design

This study was inspired from participatory design (PD) where the idea is to engage the users to innovate and develop technologies together with developers [22,23]. In a traditional PD project, the users are engaged from the beginning in defining the problem, which helps ensure that the technology meets the needs of the users. Thereafter, they are engaged in designing the technology and finally testing it [24]. In our case, we wanted to redesign the technology to meet the needs of the users, and therefore, we first explored how the users experienced problems related to their OA and rehabilitation. Then, the participants were invited to test and redesign by transforming the participants from merely informants to participants. They were asked to not just answer questions in an interview about their point of view but were also asked to actively participate in the testing and redesigning of the app, where the participants together with the researchers were *making* a mock-up of a future app [22]. The *making* of things can be a means of design participation, where the chosen tools used in the workshop allow the ability to create. The participants used their hands for expressing thoughts and ideas in the form of artifacts, which described the future app.

This study was inspired from hermeneutics philosophy, where the perspective has been to understand the participants’ lived experiences in relation to living with OA to develop a technology that meets their needs. The interpretative approach focuses on understanding experiences and on how humans make sense of their subjective reality and attach meaning to it [25,26].

### Sample and Context

#### Recruitment of Participants

The participants were recruited from Slagelse municipality (Denmark) where exercise is offered to people with OA via the Good Life with osteoArthritis in Denmark (GLA:D) project.

GLA:D is an initiative from the Research Unit for Musculoskeletal Function and Physiotherapy at the University of Southern Denmark with the overarching aim to implement current clinical guidelines for OA into clinical care. One part aims at patient education and neuromuscular exercise for patients with OA-like symptoms primarily from the hip or knee [27].

The sampling was purposive sampling. One author (AV) facilitated the contact to the supervising physiotherapist. The inclusion criterion was as follows: the participants had to have experiences with training and have an interest in using an exercise app. The exclusion criterion was as follows: people who were not able to understand Danish, as the app is in Danish.

Eight people initially agreed to participate; however, only 6 people with hip or knee arthritis participated in this study. Characteristics of the participants are shown in Table 1.

The app developer educated the participants in using the app before the test. They were giving oral and hands-on introduction to the app.

#### Roles and Relations With the Research Group

The research group was composed of both researchers with thorough experience with PD and researchers who had expertise in OA and who had been developing the app. The 3 researchers with expertise in PD were responsible for designing the different interviews and workshops, where the researcher with expertise in OA was in charge of recruitment of the participants, planning the testing of the app, and supporting the participants throughout the test.

The first author had team management skills and was responsible for the organizational and administrative procedures.

### Data Collection

**Figure 1:**
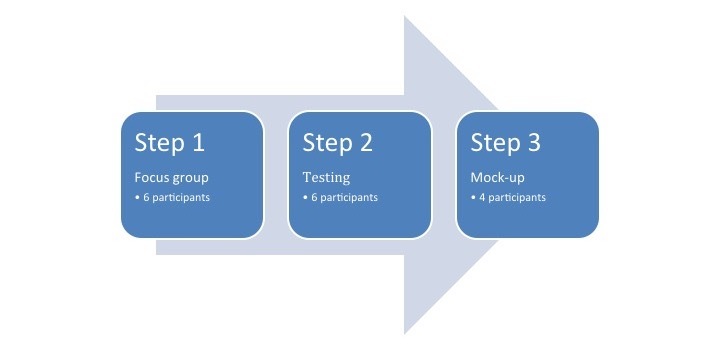
Three processes of data collection.

The data collection was divided into three processes (Figure 1).

#### Focus Group Interview

First, a focus group interview was conducted, as focus group discussions can mobilize associations *,* where the group dynamic contributes to the creation of narratives [28]. All 6 participants participated in the focus group.

**Table 1:** Characteristics of the participants.

Participant	Gender (female or male)	Ethnicity	Employment	Surgery (yes or no)	Daily exercise or activity
1	Female	Danish	Old age pensioner	No	Exercise on a daily basis
2	Male	Danish	Old age pensioner	Yes	Daily activity: walking the dog
3	Female	Danish	Employed	No	Daily activity: biking
4	Female	Danish	Employed—light duties	No	Daily activity: gardening
5	Female	Danish	—^a^	No	—
6	Male	Danish	Old age pensioner	No	Exercise on a daily basis

^a^— indicates missing data.

The focus group interview was conducted using open questions and some follow-up questions. This was to let the participants discuss freely on the topics, and allow for the possibility of asking follow-up questions, if the areas of interest for the research had not already been covered. An interview guide was compiled, and the two overall themes were on mental and physical barriers and motivational and social aspects of training at home for people with hip or knee OA. The first, second, and last authors conducted the focus group interview. It was the first author who was the primary moderator. The participants were asked how they would exercise when they were no longer part of a training class. Then, they were invited to write their reflections down, and then share them with the other group members. If needed, the moderator would ask a follow-up question and ensure that all group members shared their experiences.

At the end of the focus group, the participants were asked if they still wanted to try the training app. All participants agreed to try the app.

#### Testing of Existing App

Second, a test of the training app was initiated. The app is commercially available at App Store and Google Play Store. A user profile (email address and created password) is necessary to use the app. An underlying database holds individual user information such as exercise level, which is used for continuous individualization of exercise. The database also contains a pain and exercise diary, which is entered by the user. This allows the regeneration of the diary if it is lost from the user’s device.

The testing was divided into a set of predefined tasks or functions (eg, download the app from marketplace, create a user profile, use existing exercise programs, and maintain an exercise and pain dairy). The test period was set to last 4 weeks, and personal reminders were sent weekly on which task to focus on. Participants could contact the author (AV) for support.

To capture the participants’ experiences during the test, we used cultural probes as a mean of collecting data about their feelings and thoughts during the testing [29]. The probes are small packages that can include any sort of artifact. The package included a small notebook, a card with reflection questions for the participants, and a card with an invitation to take a photo of where and how they are trained.

#### Mock-Up Workshop

Finally, we met the participants for a mock-up workshop. Two of the participants did not participate. The purpose of the mock-up workshop was to gain knowledge of the participants’ experiences arising from testing the app, and to gain ideas for further development of the app.

**Figure 2:**
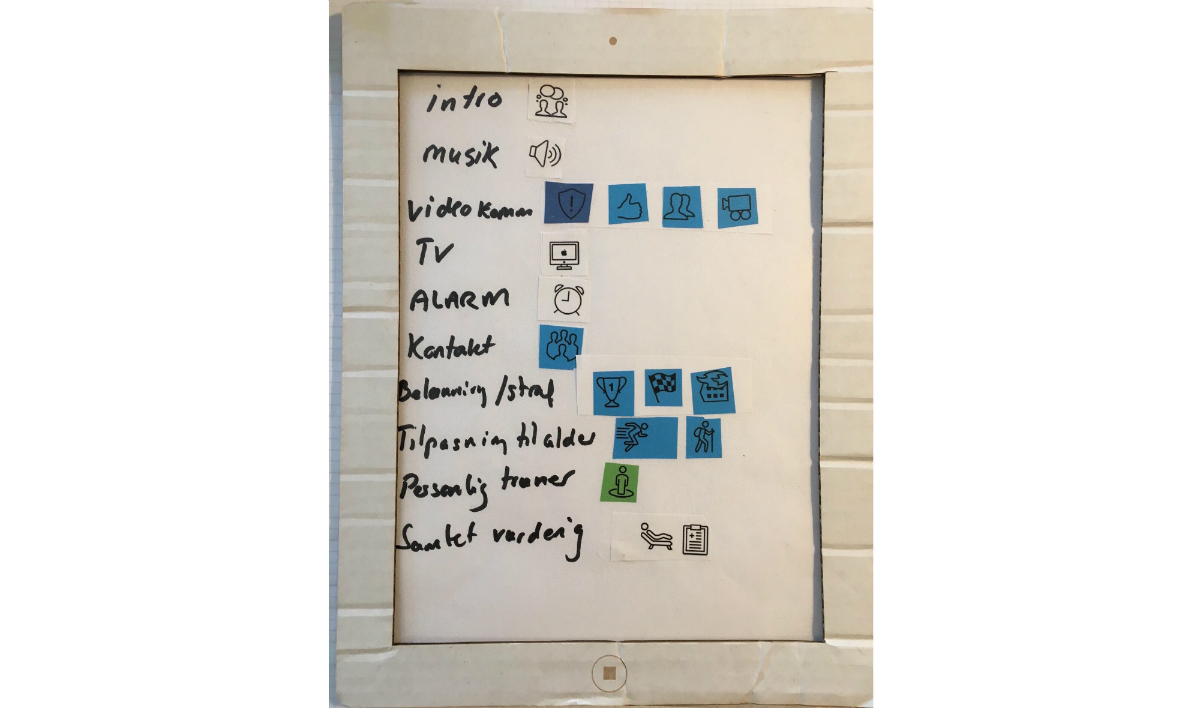
Suggestions for features in the app. The features are: introduction, music, camera, TV, alarm, award, age, personal trainer, and overall assessment.

**Figure 3:**
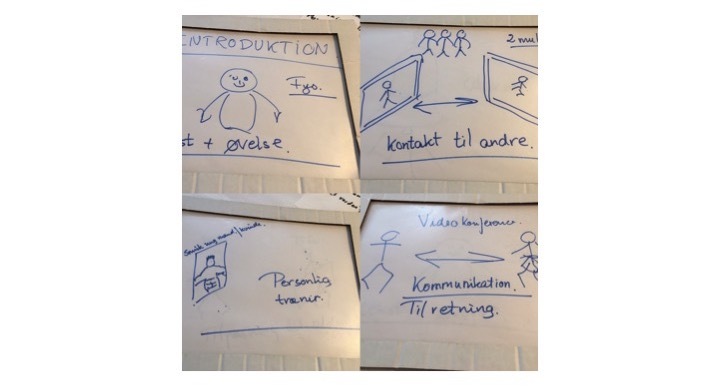
The different features reflecting the participants’ ideas about interaction.

**Figure 4:**
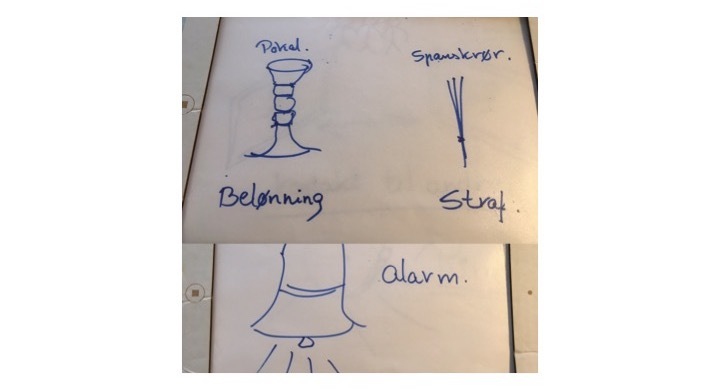
The different features reflecting the participants’ ideas about motivation and reminders.

Mock-up is a creative method where the users and the researchers together transform the users’ knowledge to solutions [30]. The starting point for the mock-up workshop was the cultural probes (from the test phase), which served as the opening to hear about the participants’ experiences. Subsequently, the participants worked on idea generation. First, ideas were written on post-it notes; then, the best ideas were chosen and the participants created a mock-up model (Figures 2-4). The participants were all active in the *making* process, and they used both language and hands for expressing their thoughts and ideas. At the end of the workshop, the participants gave feedback to the mock-up model to ensure that the result reflected the participants’ views on training.

The participants worked in a group where they had paper, colored pencils, pen, and felt-tip pens to use. The participants were introduced to the workshop for them to understand their role in designing the future app. The first, second, and last authors facilitated the workshop. It was the first author who was the primary facilitator.

Both the focus group and the workshop were audiotaped and transcribed. The focus group lasted 90 min, and the workshop lasted 120 min.

**Table 2:** Process of analysis: examples from the analysis.

Step 1: superior themes extracted after the first open reading	Step 2: From themes to codes. Identifying meaningful units. The meaningful units are coded based on the superior themes		Step 3: From codes to meaning. The meaningful units are sorted into groups
	Quotations	Code	
Competitive	*But when you can see that someone did something, then you can think to yourself that maybe I should try and do that too. Not that it is a competition but a way to challenge myself. And then I also write it down (P2)*	*Motivation*	*Competition as motivation*
Prefer being together	*I am that kind of type that feel most comfortable doing it together with someone else (P4)*	*Barrier*	*Barriers*

#### Data Analysis

The data from the focus group interview and the workshop were analyzed separately and results will be presented accordingly. The data from the focus group interview were used to identify the mental and physical barriers and motivational and social aspects of training at home for people with hip or knee OA. The data from the test and the mock-up workshop were used to get ideas for the further development of the app. The data analysis was inspired by Malterud’s systematic text condensation [31] and organized according to the steps taken in the analysis, as shown in Table 2.

First, we captured an overall impression of the data and extracted a preliminary set of main themes. Second, data were divided into meaningful topics, which were relevant to the study question. Then, the meaningful topics were condensed and coded. Finally, the findings were synthesized, involving a shift from condensation to descriptions and categories. The codes were developed based on the preliminary themes identified in the first step and the theoretical framework.

To enhance validation, the first and third authors worked on the analysis together. The analysis was performed with the transcripts printed. They discussed their overall impression of the data and then they highlighted the meaningful topics with a marker, and the codes were discussed between the 2 authors. The first author wrote down the analysis. They discussed the analysis with the other authors afterward. The findings were then discussed in relation to relevant literature and theory on motivation.

### Ethical Considerations

The participants were informed both orally and in writing about the study, and were included after providing their informed consent in compliance with the Helsinki Declaration [32].

The study was submitted to the Scientific Ethics Committee. The committee decided that approval from an ethics committee was unnecessary according to national legislation in Denmark. The Danish Data Protection Agency registered the study (2008-58-0035).

## Results

Results from the focus group interview revealed 3 categories reflecting the participants’ experiences with training and their motivation and the barriers for training.

### Results From the Focus Group Interview

#### Competition as Motivation

Most of the participants could report that competition played a central role in their motivation for exercise. They expressed that competition could be a motivational factor—not in the sense that they were competing against each other, but more a competition with yourself. It also became clear that it was important to track exercise to be able to see progress. One participant stated:

When you can see that someone did something, then you think to yourself, that maybe I should try and do that too. Not that it is a competition but a way to challenge myself. And then I also write it down.

P2

All participants were aware that each person had different challenges, and for that reason, it did not make sense to compete against each other; they were just more motivated by seeing each other’s progress and to see if they could do the same things as their peers.

#### Training Together

The majority of the participants reported that avoiding surgery was a strong motivation. One participant stated:

I found out that by doing my training, I don’t need surgery on my left knee, this keeps me going, that I don’t have to go through it once again.

P2

Most of the participants believed that training together was the main motivational factor. One of the participants stated that although she had been in pain for years and felt sorry for herself when the doctor told her that she did not need a new knee, she did not do training. It was not until she joined a training team. She stated:

I didn’t do my training, but when I joined GLA:D then I did, and it went really well. And it has given me great results […].

P3

All the 4 female participants reported that training in a group motivated them. One participant reported that she had a difficult time doing her exercises besides the supervised exercise because she needed someone to remind her *.* She stated:

But getting it done besides at the class…No it would be helpful if someone tapped my shoulder and said hey you need to do it now.

P5

All participants agreed that some kind of a reminder would be helpful; one of the participants had put elastic bands around the coffee table as a reminder to do exercise when watching television. One participant stated:

It actually means that when I sit down, I think well I can just as well do the exercises while I watch the news.

P1

#### Barriers

One of the participants reported she felt that the training helped her but could not keep the motivation when she stopped attending the class *.* She stated:

Then I stopped, and I am back into the routines of my daily life […] I have no backbone.

P3

She was not the only one who felt motivation as a barrier to do the exercises on her own.

One of the men reported that he trained by himself and his motivational factor was his daily walks with his dog *.* He stated:

I’m motivated by my dog and fresh air—training enhances my self-esteem.

P6

One of the women who had a hard time motivating herself at home also made a point that when she did not feel pain, she did not train, but if the pain came back, she would start all over *.* She stated:

I am aware of it, and if it starts to hurt again, I will do it.

P3

Another aspect that made it difficult for the participants to perform the exercise alone at home was the doubt whether they were doing the exercises correctly. The participants reported this as a reason that they stopped training. One participant stated:

I need to have some input from a professional, because it makes me aware of what I am doing and how I am moving.

P4

### Results from the Workshop

The analysis of the data from the workshop reflected how the participants had experienced using the app, their thoughts on motivation and barriers, and their ideas about creating the paper prototype.

It is presented in the following categories.

#### User Experiences

The participants shared their experiences using the app. Participants experienced different types of technical difficulties when downloading the app, or when trying to load a video—for example, the screen “freezing.” Despite these initial problems, they had all figured out how to use the app. One of the participants had sent an email to the research team, but before he had replied to her, she had found out by herself.

The participants had figured out by themselves how to use the app, but they would have liked an introduction to the app or more precisely to the exercises. One participant stated:

It looks different when you look at yourself, when you make an exercise than if you look at someone else. And to have someone correcting you. We know how important it is to perform the exercise correctly because we can make damage if we don’t do it the right way.

P4

They all stated that a physical meeting before starting using the app is necessary, but they also discussed that the app could be the follow-up offer after their participation at the GLA:D class ended. One participant stated:

I think that if we had gotten the app earlier, and we could have used it as a continuation of the class, then it had been easier and I would have continued with the training, and then we could also have had the physical therapist words in the back of our minds.

P3

It was also reported that instead of using the app, they had used the exercise instructions on paper sheets that was handed out at the class because it was familiar and they felt confident in the familiarity of the exercises.

They all agreed that they missed the class, and they would have liked something after the classes stopped. One of the participants had written the following in her notebook (handed out as part of the cultural probe):

You wrote the question: “do you miss being in the GLA:D class?” And I wrote “The class made me feel obligated to go. Now it is easier to find excuses not to do the training. And what I miss the most is the professional guidance when we were together with the physical therapists.

P3, note in notebook, cultural probe

#### Motivation

One of the participants saw that the app could help her in different ways, both to get the actual exercises done and also to register different activities such as biking, running, and gardening. She needed to lose weight and saw it as a way to keep track of her activity level. However, she pinpointed how the social aspect of being in a class was important for her training and thereby her rehabilitation. She stated:

The motivation is there, but then again—it is in an app, and I am better in the class. And there I remember that I’m not the only one who feels that way.

P3

The participants reported that the app was easy to use, which they found motivating. They also found that the exercises were good. However, they all agreed that they needed something more that could make them stick to the training. One of the participants had been involved in a project where she should write in a diary how she felt and experienced the trainingand rehabilitation. Every month, she received a message if she had not done it. She was motivated by that kind of monitoring.

The participants also discussed that although they ought to take responsibility for their own health, it had a positive effect if they knew someone else was “watching.” She stated:

It is a great help that someone is saying “hello’’ (she knocks the table to underline the meaning).

P1

#### Ideas for a Prototype

The participants made a mock-up of how the app should look to meet their needs and thereby addressing their barriers/enablers that were identified in the focus group interview. The following section is a description of their ideas, and some of their drawings are selected to show how they worked with designing the paper prototype.

Figure 2 shows the suggestions for features in the app. The features are: introduction, music, camera, television, alarm, award, age, personal trainer, and overall assessment.

The ideas were categorized into the following: interactive, motivation, and user experiences.

##### Interactive

The participants requested an introduction to the app. It could be at a physical meeting with a physiotherapist or via a feature in the app. They suggested that it could be a visual guidance to the different exercises supplemented with text. In addition, they asked for the possibility to have video calls where they could get advice and have their exercises corrected.

It was also important to the participants that they could have contact with other patients, as it was highlighted during the first focus group interview that the participants were motivated by training together with others and by seeing each other’s progress and to see if they could do the same things as their “class mates” or peers. This could, for instance, be a chat forum where they could share their experiences with training.

##### Motivational features

The participants had several suggestions for different motivational features. For instance, they could either get rewarded or be given a “penalty.” In addition, they suggested that the app should have an alarm where they were notified that it was time to exercise. This could also meet the needs of the majority of the participants who in the focus group interview reported that they needed someone to remind them.

##### Individualized features

The participants wished for more individualized features to make the user experience better. For instance, they would like to be able to choose music. They would also like to have the exercises shown on their television.

## Discussion

### Principal Findings

OA is a chronic and degenerative joint disease, which causes pain and decreased physical function. Exercise has proven effective in diminishing pain and to postpone the need for surgical intervention. However, the lack of continuous adherence to exercise remains a challenge for people with OA as well as patients with other chronic diseases and obesity.

The results from this study revealed that all the participants wanted to do their training and knew that it was the best way to avoid operation and to minimize their pain. Hence, half of the participants described being motivated on one hand to avoid operation and unnecessary pain, but others found it hard to motivate themselves to do the exercises on their own. They missed the observation, comments, and encouragement by the supervising physiotherapist as well as their peers. They described motivation in the exercise community as the feedback from the instructor, and they were also depending on “the tap on the shoulder” to get going. Nevertheless, some of the patients created their own tailored activities and included these in their daily life, which became a driving factor for sustainable motivation.

The participants made a mock-up of how the app should be redesigned to meet their needs and thereby addressing their barriers or enablers that existed.

### Motivation—Internal and External Facilitators and Barriers

Motivation was a major issue for the participants. Motivation is the element within the individual, which evokes and maintains certain behaviors [33]. Motivation can shed light on the reasons for someone to act in a certain way [34]. Different things motivated the participants in our study. Among other things that motivated them, they highlighted competition (both with themselves and each other), being social, avoiding pain, avoiding surgery, and being able to walk the dog. These different things can be characterized as *internal* and *external* motivation [33]. External motivation is created from outside the person, and cannot be controlled by an individual. Internal motivation is created within the individual, and the behavior occurs, as it is satisfying for the person. An important aspect of the internal motivation is a feeling of being *capable*. This can be enhanced by experiences of success when training. In this study, all participants highlighted the importance of contact with the therapist to be corrected in the way of doing the exercises and at the same time getting reassurance from the expert, and it was also reflected in the redesigning of the app. This may have to do with the participants not feeling competent while doing the exercises alone. Feeling competent may increase the participants’ motivation to training. Similarly, feeling unsuccessful can weaken their motivation. This can be linked to the concept of self-efficacy. Self-efficacy refers to the belief in one’s ability to successfully perform a particular behavior [35]. Bandura’s theories originated in behaviorism but took a more humanistic approach, with a focus on the social, biological, and cognitive aspects of learning. Bandura’s social learning theory involves the concept of self-efficacy. Bandura states that self-efficacy beliefs influence the way that people think, feel, and act [35]. For patients to positively engage exercise behavior, they must have confidence in performing the specific behavior. Patients with high self-efficacy are likely to make a greater effort than patients with low self-efficacy. According to Bandura, perceived self-efficacy plays a key role in adapting to the new behavior. Self-efficacy beliefs are built either through one’s own experiences (mastery experiences), other experiences (vicarious experiences), support from people in one’s environment (verbal persuasion), or through emotional experiences (physiological and affective state of mind).

According to Bandura, self-efficacy beliefs should incorporate the level of specific knowledge pertaining to the actions involved in training as well as confidence in one’s ability to carry out the specific activities [36]. Previous experiences, both positive and negative, as well as a lack of experiences, have an impact on patients’ perceptions of efficacy. Psychosocial mood also has an influence on the experiences. A positive attitude toward training, a good experience of training, and a positive state of mind also positively affect training experiences [36].

Vicarious experiences, as well as social and verbal persuasion, from family, peers, and therapists contribute to self-efficacy. This underlines the importance of both having good experiences with training, as well as the possibility of training together, and the feedback from a therapist [35,36]. It was also highlighted in the redesigning of the app where the participants requested a chat room, where they could connect with peers.

Petursdottir et al [37] identified facilitators and barriers, which will influence a person with OA exercise behavior. The internal facilitators and barriers are both personal experiences such as effect on pain, finding suitable exercise, and the benefits of exercising. This was also found in this study—the 2 men who participated were both motivated by internal facilitators, whereas the 4 women were all motivated by the external facilitators. The external facilitators described by Petursdottir et al were the physiotherapist’ professional care, training partners, and the availability of exercise classes.

Pertursdottir et al found the effect on pain to be the most significant factor. In this study, the participants also emphasized this as an important facilitator; however, the participants could also report that when the pain was gone, it was hard for them to continue training, although they knew that there was a risk that the pain would return.

The participants also underlined that it was difficult to keep up with the training after stopping the supervised class because they got out of the training routine and fell into their daily routine, and the participants also expected that the technology should be able to help them to keep up with a daily training routine both by having a reminder function and the possibility to connect with other peers. This can be explained by the literature, where other studies found that an important factor for the patients to do the exercises is being part of an organized training activity [37,38].

### Exercise Community

The majority of the participants stressed that the company of others motivated them and that the training was conducted in a class and with guidance from a therapist. This is found in other studies as well, where social support is highlighted as a significant motivational factor [37,39,40].The support can be from the person’s family and friends, as well as health care professionals, or from a training class.

The majority of the participants were depending on the social support, and although they all agreed that they had the main responsibility themselves, they were motivated if someone kept them on a short leash. This can be explained with a behavioristic view of the changing behavior, where changing behavior occurs with either a reward or penalty. As well with the concept of self-efficacy, where learning from others, play an important role in building your self-efficacy [35]. This is essential to consider when designing a training app.

### Technology as a Way to Overcome the Barriers

Other studies have shown how the use of interactive technology can motivate participants. Thorup et al found that a pedometer could offer independence from standardized rehabilitation as it could individualize the walking activity based on the patient’s choice. The pedometer delivered feedback on walking activity, which led to an increased competence for the patients to achieve their goals for steps [41]. This can be linked to the motivational theory, where feeling competent while doing the exercises can support the participants’ motivation [33].

Thorup et al also found that the pedometer supported relatedness with others. The health professionals’ surveillance of patients’ steps made the patients feel observed and supported [41]. This is interesting in regard to our findings, where the participants were depending on the social and interactive aspects of training, which was reflected in their demands for the redesigning of the app. This is a way in which the app can be further developed, thereby overcoming the barriers identified.

There is no evidence published on whether technologies can influence patients with OA training and their outcomes. There are some studies showing that consistent contact via phone can improve the clinical status of patients with knee OA [7], whereas a randomized controlled trial revealed that monthly phone contact aimed at promoting self-care for patients with knee OA could be associated with improvements in joint pain and physical function [42]. This is consistent with the findings of our study, where the personal contact is an external motivation for the participants. This supports the participants’ ideas for interactive elements in the app, and also their experiences using the app, where they found it easy to use, and they also found that the exercises were good, but it was not enough for them to use it. The participants had ideas to support the experience of “going to class” and to get feedback from a therapist.

The idea of being *controlled* and receiving reminders, as well as rewards or penalty, corresponds well with the use of automated interactive technology.

One of the most usual apps is a reminder for patients to take their medications with the use of text messaging [43]. In addition, there is the potential that redesigning the app with a reminder function can encourage the participants’ training behavior. As shown in the study of the pedometer, the surveillance of patients’ steps made the patients feel observed and supported and thereby encouraged them to do the exercises [41,44]. One of the participants explained how she had customized her coffee table for exercise with the use of elastic bands, so she could do her training while watching the news. It shows how small adjustments to the daily routines can change behavior. This can be captured in a training app, where regular messages are customized for the individual to enhance training.

### Strengths and Limitations

The limitation of this study is that it was a small-scale study; however, most qualitative studies are typically small-scale.

Therefore, despite the small sample size, the aim of this study, as other qualitative studies, was to provide in-depth exploration of the phenomenon under investigation. Therefore, the intention of this study was to understand and explain the mental and physical barriers and motivational and social aspects of training at home for people with hip or knee arthritis and to enhance and test an exercise app for use at home by patients with hip or knee OA.

However, it has been taken into account that only 6 patients with OA from a specific medical center or community center were included, and a wider representative sample would have provided more in-depth information. Future studies may have to consider this.

We have provided rich descriptions of both the mental and physical barriers and motivational and social aspects of training at home for people with OA, as well as rich and visual descriptions of their experiences with an exercise app and also the suggestions for further development of the app.

This will hopefully allow the readers to judge whether the work is potentially transferable to their own contexts. The results cannot claim statistical generalizability, but analytical generalization [45], which emerges by means of the dialectic between theory and practice.

The analysis was conducted together with coresearchers to increase the reliability, and we presented the analysis process in a table to make the analysis transparent. Quotations from the focus group interview were used to link to the participants’ original statements to warrant validity.

### Conclusions

The conclusion of the study is that the long-term continuation of exercising for patients with OA could be improved with the use of a technology tailored to users’ needs, including motivational and other behavioral factors. The study highlighted that the continuation of rehabilitation is easiest in the short term when the benefit for the patient is visible and rewarding. In the long term, it takes more motivation to continue—motivation was often facilitated by a physical meeting with the therapist. We need to find new ways of connecting the therapist and peers to the patient’s daily life, and health technology as a tailored app seems to hold promises.

The participants were informed both orally and in writing about the study, and were included after providing their informed consent in compliance with the Helsinki Declaration [32].

The study was submitted to the Scientific Ethics Committee. The committee decided that approval from an ethics committee was unnecessary according to national legislation in Denmark. The Danish Data Protection Agency registered the study (2008-58-0035).

## Abbreviations

GLA:D: Good Life with osteoArthritis in Denmark
OA: osteoarthritis
PD: participatory design
Ther-ex: Therapeutical exercise
